# Proteolysis activation of Cry1Ac and Cry2Ab protoxins by larval midgut juice proteases from *Helicoverpa armigera*

**DOI:** 10.1371/journal.pone.0228159

**Published:** 2020-01-31

**Authors:** Shaoyan Liu, Shuo Wang, Shuwen Wu, Yidong Wu, Yihua Yang

**Affiliations:** College of Plant Protection, Nanjing Agricultural University, Nanjing, Jiangsu, China; Chinese Academy of Agricultural Sciences, CHINA

## Abstract

Proteolytic processing of *Bacillus thuringiensis* (Bt) Cry protoxins by insect midgut proteases is critical to their insecticidal activities against target insects. Although transgenic Bt cotton expressing Cry1Ac and Cry2Ab proteins have been widely used for control of the cotton bollworm (*Helicoverpa armigera*) in the field, the proteolytic cleavage sites in the two protoxins targeted by *H*. *armigera* midgut proteases are still not clear. In this study, the proteolysis of Cry1Ac and Cry2Ab protoxins by midgut juice prepared from midgut tissue of *H*. *armigera* larvae was investigated. Cleavage of Cry1Ac protoxin by midgut proteases formed a major protein fragment of ~65 kDa, and N-terminal sequencing revealed that cleavage occurred at Arg28 in the fore-end of helix α-1 in domain I of Cry1Ac. Cleavage of Cry2Ab protoxin by midgut juice proteases produced a major protein fragment of ~50 kDa, and the cleavage occurred at Arg139 between helices α-3 and α-4 in domain I of Cry2Ab. The amino acids Arg28 of Cry1Ac and Arg139 of Cry2Ab were predicted as putative trypsin cleavage sites. Bioassay data showed that the toxicities (LC_50_s) of Cry1Ac and Cry2Ab protoxins were equivalent to those of their respective midgut juice-activated toxins in the susceptible SCD strain of *H*. *armigera*. Identification of the exact sites of N-terminal activation of Cry1Ac and Cry2Ab protoxins will provide a basis for a better understanding of the mode of action and resistance mechanisms based on aberrant activation of these protoxins in *H*. *armigera*.

## Introduction

*Bacillus thuringiensis* (Bt) is a ubiquitous gram-positive bacterium, and during sporulation, Bt strains produce crystal proteins (Cry toxins) that are toxic to a variety of insects, such as lepidopterans, coleopterans, dipterans and hemipterans [1]. Bt Cry toxins have been extensively used in sprays and transgenic plants, which has contributed to the efficient control of different agricultural pests. They also have reduced the use of chemical insecticides and increased farmer profits [2–5].

The majority of Cry toxins are produced in insoluble and inactive forms as crystal inclusions composed of protoxins. After ingestion by target insect larvae, the crystals are solubilized in the alkaline environment of the larval midgut and are activated by midgut proteases [6]. Then, the activated toxins pass through the peritrophic matrix and sequentially bind to specific receptors located on the brush border membrane (BBM) surface of the cells, leading to membrane insertion and pore formation [7,8]. It is generally accepted that the activation of protoxins is one of the most important and essential steps to exert toxicity [9–11].

Considering the molecular weight of Cry proteins, two types of protoxins have been identified: short protoxins of approximately 70 kDa (such as Cry2Ab) and long protoxins of 130 kDa (such as Cry1Ac) [9]. In the case of the short protoxins, approximately 40 amino acids of the N-terminal end are removed during activation with midgut proteases, while for the long protoxins, in addition to N-terminal processing, this activation entails removal of 500–600 amino acids from the C-terminal end. Both cases result in activated Cry toxins of ~60 kDa [1,9,12]. The midgut proteases of lepidopteran larvae mainly belong to the serine protease class, such as trypsin-like and chymotrypsin-like proteases [13–15]. Such midgut proteases are likely to be responsible for protoxin activation. It was reported that improper activation, such as insufficient processing or over digestion, in some insect populations has resulted in insect resistance to Cry protoxin action [16].

The cotton bollworm, *Helicoverpa armigera* (Hübner), is one of the most invasive pests infesting cotton, maize and other crops. This insect originated from Africa, Asia, Europe and Australia; however, long-range migration and international trade helped this pest spread throughout South and Central America [17,18]. In China, the planting of transgenic cotton expressing only Cry1Ac since 1997 has been very successful in controlling *H*. *armigera* [19,20]. Although Bt cotton has remained useful against *H*. *armigera*, regular and significant increases in the frequency of resistant individuals to Cry1Ac have provided an early warning for the possibility of resistance evolution in northern China [21,22]. The amino acid sequence identity of Cry2A with Cry1Ac and other Bt toxins is less than 20% [23], so Cry2A exhibits a lower level of cross-resistance with other Bt toxins, such as Cry1Ac [24,25]. Therefore, to delay the evolution of resistance, Bt cotton pyramids that produce the Cry1Ac and Cry2Ab toxins have been successfully planted in the United States, Australia and India [26].

Although Cry1Ac and Cry2Ab toxins have been widely used to control *H*. *armigera*, their proteolytic cleavage sites targeted by *H*. *armigera* midgut proteases are still not clearly defined. In the present work, we investigated the proteolysis of Cry1Ac and Cry2Ab protoxins by *H*. *armigera* midgut juice *in vitro* and detected the proteolysis by SDS-PAGE. Verification of N-terminal sequences of the activated toxins, ~65 kDa (for Cry1Ac) and ~50 kDa (for Cry2Ab), by Edman degradation sequencing analysis showed that the proteolysis of Cry1Ac and Cry2Ab protoxins occurred at Arg28 and Arg139, respectively. Determination of the cleavage sites provided a basis for further study of the mechanism of action and resistance caused by abnormal activation.

## Materials and methods

### Insect strain

The susceptible strain (SCD) of *H*. *armigera* was collected from the Ivory Coast, Africa, in the 1970s and has been maintained in laboratory conditions without exposure to Bt toxins or other insecticides for more than 40 years [27]. Larvae were reared on an artificial diet based on wheat germ and soybean power at 26 ± 1°C with a photoperiod of 16:8 h (Light:Dark).

### Preparation of Bt toxins

Cry1Ac protoxin was produced from the HD-73 strain of *B*. *thuringiensis* subsp. *kurstaki*, and Cry2Ab protoxin was produced from the engineered HD-73^-^ strain containing the *cry2Ab* gene. The Bt strains were grown for 36 h at 30°C in lysogeny broth (LB) medium until 50% of the crystal was released. The crystals were collected by centrifugation at 5,000 rpm for 20 min, and then the pellet was washed with sterile water, followed by 1 M NaCl. The crystals were dissolved in 100 mM Na_2_CO_3_ buffer (pH 10.5, containing 10 mM DTT). Purified Cry1Ac and Cry2Ab protoxins were checked by SDS-PAGE. The protein concentrations were determined by the method of Bradford [28], using bovine serum albumin (BSA) (Solarbio, Beijing, China) as a standard.

### Midgut juice preparation

Fifth instar 2^nd^ day larvae from the susceptible SCD strain were chilled on ice for 10 min before they were dissected. Ten midguts were dissected, and midgut tissues were separated from peritrophic membranes containing the food bolus. The content was homogenized with 2 ml of cold NaCl solution (0.15 M) and centrifuged at 12,000 rpm for 15 min at 4°C; the clear supernatants were collected. The protein concentration of midgut juice was quantified by the method of Bradford [28] and adjusted to 1 mg/ml; the stocks were then frozen at -80°C until use.

### Digestion of Cry1Ac and Cry2Ab protoxins by midgut juice

Before use, the two protoxins were diluted to 0.5 mg/ml, and the 1 mg/ml midgut juice was diluted to six different concentrations. Ten microliters of each protoxin (5 μg) were mixed with 2 μl of six different concentrations of midgut juice (500, 250, 50, 25, 12.5, 2.5 μg protein/ml) in a final volume of 20 μl of 100 mM Na_2_CO_3_ buffer (pH 10.5). The mixtures were incubated at 37°C for 1 h. The reactions were stopped by adding 1 μl of 10 mM PMSF. The samples were separated by 10% SDS-PAGE and stained with Coomassie blue R-250 for 1 h. The proteins were visualized by destaining with 10% acetic acid.

### N-terminal sequencing

Activated Cry1Ac and Cry2Ab toxins were prepared by incubating 1:50 (w/w) midgut juice/protoxin for 1 h at 37°C. A total of 30 μg of each protoxin was incubated with midgut juice; the reactions were stopped by adding 5×SDS-PAGE sample buffer and heated for 5 min at 95°C. Then, the samples were divided into six replicates and separated by 10% SDS-PAGE. After electrophoresis, the proteins were transferred to PVDF membrane (Bio-Rad, Hercules, CA, USA) using CAPS buffer (10 mM CAPS, 10% methanol, pH 11.0) at 350 mA (constant current) for 1 h at 4°C. Before staining, the PVDF membranes were soaked in methanol solution for 15 s, stained with 0.1% (w/v) biological dye ponceau S (containing 5% acetic acid) for 10 min, and washed with distilled water until the red background faded completely. The activated toxin bands were clearly marked and submitted for N-terminal sequencing based on Edman degradation using a Shimadzu automated protein/peptide sequencer (PPSQ-333A, Kyoto, Japan).

### Bioassays

Cry1Ac and Cry2Ab midgut juice-activated toxins were prepared by incubating 20:1 (w/w) of protoxin:midgut juice at 37°C for 1 h. The toxicological responses of Cry1Ac and Cry2Ab protoxins and midgut juice-activated toxins were determined against neonates of the susceptible SCD strain of *H*. *armigera* with diet surface overlay bioassays as described previously [29]. Gradient concentrations of Bt protein solution were prepared by diluting the stock suspensions with 0.01 M phosphate buffer solution (PBS), pH 7.4. A liquid artificial diet (900 μl) was dispensed into each well of a 24-well plate. After the diet cooled, 100 μl of Bt protein solution was applied evenly to the diet surface in each well. A single unfed neonate (24 h old) was placed in each well, and mortality was recorded after 7 days. Forty-eight larvae were tested for each concentration. Larvae were considered dead if they died or weighed less than 5 mg at the end of bioassays. The LC_50_ values (the concentration of Bt protein that killed 50% of larvae) and the 95% fiducial limits of the LC_50_ for each strain were calculated through probit analysis of the mortality data using the PoloPlus program [30].

## Results

### Proteolysis of Cry1Ac and Cry2Ab protoxins and N-terminal sequencing

**Fig 1:**
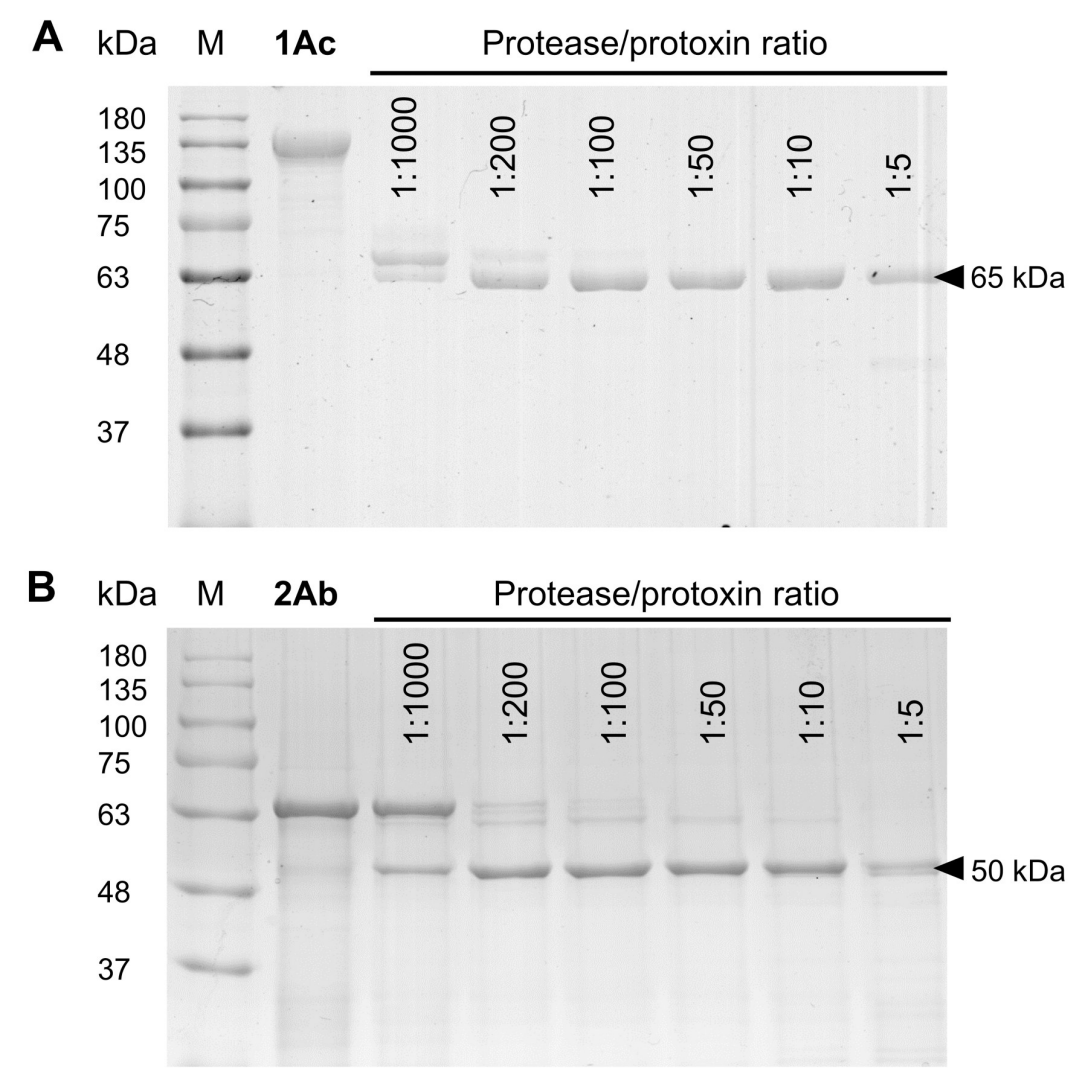
SDS-PAGE analysis proteolytic digestion of protoxins by *H*. *armigera* larval midgut juice. (A) The digestion of Cry1Ac protoxin. (B) The digestion of Cry2Ab protoxin. M, the BlueRay prestained protein marker (MDBio, Qingdao, China). Both protoxins were incubated with midgut juice at different midgut juice protein/protoxin ratios (total protein content of the gut juice added/5 μg protoxin protein) for 1 h at 37°C.

Protoxins were digested with *H*. *armigera* midgut juice at 37°C using different midgut juice/protoxin ratios. Analysis of SDS-PAGE showed that digesting Cry1Ac protoxin with low concentrations of midgut juice resulted in a doublet band of ~70 and ~65 kDa. As the ratio of midgut juice/protoxin increased, the ~70 kDa protein was completely converted to ~65 kDa, which remained stable until the concentration of midgut juice increased to 1:5. Slight degradation was observed (Fig 1A). For Cry2Ab protoxin, at low midgut juice concentrations, only a small amount of protoxin was converted to an ~50 kDa protein. As the ratio of midgut juice/protoxin increased, the amount of Cry2Ab protoxin significantly decreased until all protoxin was converted to activated toxin (Fig 1B).

**Fig 2:**
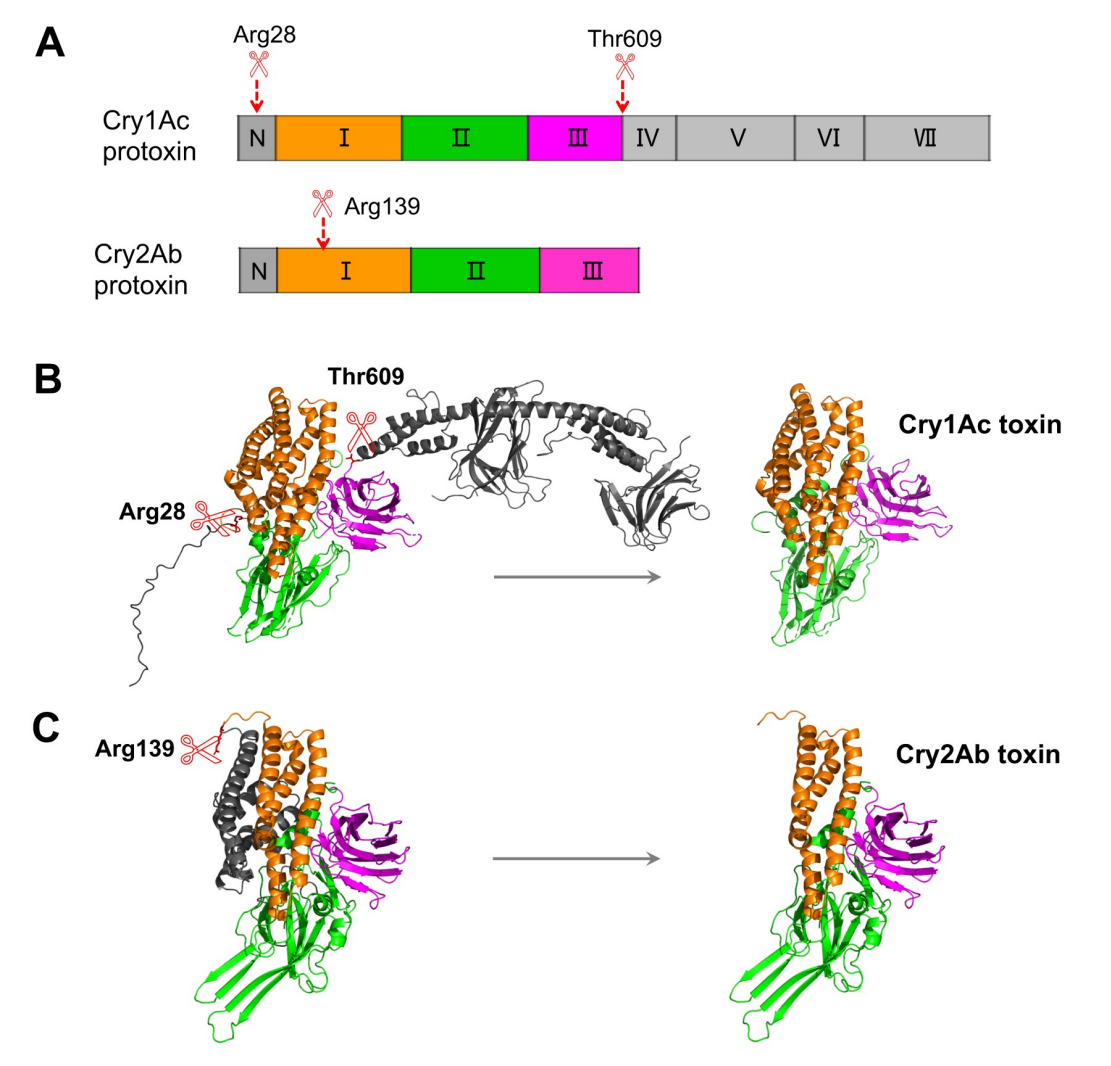
The structure of Cry1Ac and Cry2Ab. (A) The schematic diagram of Cry1Ac and Cry2Ab protoxins. The red dotted arrows represent cleavage sites. (B) Protein 3D structures of Cry1Ac protoxin and activated toxin. (C) Protein 3D structures of Cry2Ab protoxin and activated toxin. The three-dimensional structures of Cry1Ac and Cry2Ab protoxins were based on homologous modeling, built with SYBYL-Orchestrar software and viewed by the PyMOL program. The removed parts are shown in gray. The orange, green and purple represent domains I, II and III, respectively. Arg28 and Thr609 represent the cleavage sites of protease hydrolysis of Cry1Ac protoxin from the N- and C-termini, and Arg139 represents the cleavage sites of Cry2Ab from the N-terminus.

Ten cycles of Edman degradation reactions were executed to determine the N-terminal sequences of the activated Cry1Ac and Cry2Ab toxins. The amino acid sequences of the N-terminal end indicated that proteolysis by the midgut juice proteases of *H*. *armigera* cleaved Cry1Ac protoxin at Arg28 and Cry2Ab protoxin at Arg139 (Fig 2A); both residues are predicted as putative trypsin protease cleavage sites.

### Structural analysis of Cry1Ac and Cry2Ab protoxins and toxins

The 3-D Cry toxin family is the largest group of Cry toxins, and they are globular molecules containing three structural domains. As mentioned before, two types of 3d-Cry protoxins have been identified: Cry2Ab is a short protoxin, while Cry1Ac is a long protoxin. Although Cry1Ac and Cry2Ab share low amino acid sequence identity, they share a similar fold consisting of three domains. In addition to the three-domain toxic core, the Cry2Ab protoxin has an additional N-terminal end (Fig 2A). Domain I is a bundle composed of seven α-helices; our data indicate that digestion with the midgut proteases of *H*. *armigera* introduced a cleavage between helices α-3 and α-4 in domain I. This suggests that the region comprising the N-terminus and helices α-1 to α-3 of Cry2Ab was removed in the activated toxin (Fig 2C). However, the denaturant conditions of the SDS-PAGE analysis do not allow us to conclude if this region is completely detached from the protein or remains attached by noncovalent interactions. Compared with the structure of Cry2Ab protoxin, Cry1Ac protoxin has an additional long C-terminal end composed of four discrete domains (domains IV-VII) (Fig 2A). The activation of Cry1Ac cleaved the entire C-terminal region and 28 amino acids from the N-terminus in the fore-end of helix α-1 in domain I (Fig 2B), yielded an ~65 kDa activated toxin. Again, we want to state that these data would not allow us to conclude if some peptides from protoxin still remain attached to the activated toxin. More studies will be needed in the future to clearly demonstrate if activation is linked to protein degradation of the resulting peptides.

### Toxicity of the protoxins and digested toxins to *H*. *armigera*

The LC_50_ values for Cry1Ac protoxin and midgut juice-activated toxin against the SCD strain were 0.0056 and 0.0063 μg/cm^2^, respectively, and their 95% fiducial limits overlapped (Table 1), indicating that the toxicities of the two forms of Cry1Ac were not significantly different. The LC_50_ value for Cry2Ab protoxin (0.10 μg/cm^2^) was roughly equivalent to that of midgut juice-activated toxin (0.19 μg/cm^2^), although their 95% fiducial limits did not overlap (Table 1). As expected, the toxicities of Cry1Ac were approximately 20-fold higher than that of Cry2Ab to the SCD strain of *H*. *armigera*.

**Table 1:** Toxicity of Cry1Ac and Cry2Ab protoxins and their respective midgut juice-digested toxins against neonates of the susceptible SCD strain of *H*. *armigera*.

Bt toxin	N^a^	Slopes ± SE	LC_50_ (μg/cm^2^)	95% FL^b^
Cry1Ac protoxin	336	1.46 ± 0.16	0.0056	0.0036–0.008
Cry1Ac toxin	336	1.58 ± 0.16	0.0063	0.0048–0.008
Cry2Ab protoxin	336	2.39 ± 0.21	0.10	0.08–0.12
Cry2Ab toxin	336	1.69 ± 0.17	0.19	0.15–0.25

^a^Total number of individuals tested.

^b^95% fiducial limits of LC_50_.

## Discussion

All 3d-Cry proteins are produced as protoxins, and the proteolysis of protoxins by midgut proteases to produce the activated toxin core is crucial to exert toxicity [8,9]. Here, we demonstrated that proteolytic processing of Cry1Ac and Cry2Ab protoxins with *H*. *armigera* midgut juice resulted in activated toxins of ~65 and ~50 kDa, respectively (Fig 1). For Cry1Ac-activated toxin, the N-terminal sequences indicated that the first 28 amino acids of the N-terminal end before helix α-1 in domain I were cleaved, while for Cry2Ab-activated toxin, cleavage occurred at Arg139, which is located in the loop between helices α-3 and α-4 in domain I (Fig 2). Determination of the cleavage sites would provide a basis for further study of the mechanism of action, and these data are helpful for investigating insect resistance mechanisms caused by abnormal proteolysis of these Cry protoxins.

In our work, we found that Cry1Ac protoxin was digested by *H*. *armigera* midgut proteases, finally yielding an ~65 kDa activated toxin, which was resistant to further proteolysis within a wide range of protease concentrations (Fig 1A). The N-terminal sequence of this product indicated that proteolysis occurred at Arg28 in the fore-end of α-1 in domain I (Fig 2B), sharing the same cleavage site that was described with commercial trypsin. Digestion of Cry1Ac protoxin by midgut proteases isolated from *Pieris brassicae* and *Mamestra brassicae* both produced a soluble form of the toxin with a molecular mass of ~60 kDa. N-terminal sequencing of these products indicated that proteolysis occurred at Arg28 in both cases [31]. Similarly, the same cleavage site was found after treatment with midgut juice from *Adoxophyes* spp. and *Bombyx mori* [32]. In contrast, midgut juice proteases that are present in *Plutella xylostella* larval midgut digested Cry1Ac protoxin at Leu46, located after helix α-1, while proteolytic activation of Cry1Ac by *Spodoptera litura* midgut proteases cleaved the protein at Gly66, located before helix α-2b [32].

Proteolytic activation of Cry2Ab protoxin by midgut juice of *H*. *armigera* resulted in an ~50 kDa protein (Fig 1B). Proteolysis occurred at residue Arg139, which is located on the loop structure between helices α-3 and α-4 in domain I (Fig 2C), and this site was predicted as a putative trypsin cleavage site. When Cry2Ab protoxin was cleaved by *P*. *xylostella* midgut proteases, a single band of 50 kDa was formed. Interestingly, further analysis showed that this single band was a mixture of two proteins, and the N-terminal sequences demonstrated that cleavages occurred at Arg139 and Leu144, predicted as trypsin and chymotrypsin cleavage sites, respectively [33]. The Cry2Ab and Cry2Aa amino acid sequences showed 87% identity [34], and the diversity in amino acid sequences indicated that the proteolysis of Cry2Ab might differ from that of Cry2Aa. Previously, cleavage of the Cry2Aa1 and Cry2Aa3 protoxins by *Lymantria dispar* and *B*. *mori* larvae midgut juice produced two major bands, 58 and 49 kDa toxins. N-terminal sequencing confirmed that the first cleavage site was at Tyr49, which is on the loop between α-0 and α-1, while the second cleavage site was at Leu144, which is on the loop between α-3 and α-4 [35,36].

It is believed that the mode of action of Cry toxins in lepidopteran starts with proteolytic activation by midgut proteases, the correct activation of the protoxin is an essential step for proper insecticidal activity [10], and low susceptibility may be caused by improper processing of Cry toxins [16]. It has been reported that a mutant Cry1Ac protoxin that affects its proteolytical processing at the N-terminal end by *Manduca sexta* midgut juice resulted in 25-fold lower toxicity than the properly activated toxin lacking the first 28 amino acids [10]. A major midgut protease (T1) that activated Cry1A protoxins was found to be absent in the midgut extracts from the 133-r and 198-r strains of *Plodia interpunctella* that are resistant to Cry1A action, and it was confirmed that the absence of T1 was genetically linked to Cry1A resistance [37]. Another protease-mediated Cry1Ac resistance mechanism was found in a *Heliothis virescens* strain with reduced protoxin processing [38]. Additionally, a laboratory-selected strain of *H*. *armigera* showed 72-fold resistance to Cry1Ac protoxin; the larval midgut juice from this resistant strain displayed improper processing of the protoxin, which was likely caused by the down regulation of *HaSP2* protease [39]. In addition, 99% reduced transcription of the serine protease *HaTryR* was reported to be related to Cry1Ac resistance in *H*. *armigera* [40]. Clearly, almost all of the results described above indicated that protease-mediated resistance to Cry toxins was associated with at least one major serine protease. However, the composition of insect midgut proteases is highly variable, and the proteolytic activation of Cry protoxins is a complex process. Therefore, the role of each protease in this process is difficult to predict. Moreover, there has been no conclusive evidence that *in vivo*, a single midgut protease can activate Cry protoxins alone.

Previous studies have shown that Cry1Ac and Cry2Ab do not share receptors in *H*. *armigera* [41–43]; thus, it is believed that there is a low risk of cross-resistance between the two toxins, which is mainly based on receptor alterations. However, if a single or few common proteases account for the activation of both Cry1Ac and Cry2Ab protoxins, there may be a risk of cross-resistance between these two toxins. In the future, it is necessary to identify the exact midgut proteases of *H*. *armigera* that are responsible for activation of Cry protoxins and determine whether few specific proteases or a pool of proteases participate in this process.
